# Combination of VP3 and CD147-knockdown enhance apoptosis and tumor growth delay index in colorectal tumor allograft

**DOI:** 10.1186/s12885-016-2530-8

**Published:** 2016-07-13

**Authors:** Ruzila Ismail, Zeenathul Nazariah Allaudin, Rasedee Abdullah, Mohd-Azmi Mohd Lila, Nik-Mohd-Afizan Nik Abd. Rahman, Sheikh-Omar Abdul Rahman

**Affiliations:** Laboratory of Immunotherapeutic and Vaccines, Institute of Bioscience, Universiti Putra Malaysia, 43400 Serdang, Selangor Malaysia; Department of Pathology and Microbiology, Faculty of Veterinary Medicine, Universiti Putra Malaysia, 43400 Serdang, Selangor Malaysia; Department of Veterinary Laboratory Diagnosis, Faculty of Veterinary Medicine, Universiti Putra Malaysia, 43400 Serdang, Selangor Malaysia

**Keywords:** pVIVO1-GFP/VP3, psiRNA-CD147/2, CT26 colon cancer cell tumor, Apoptosis

## Abstract

**Background:**

Cancer therapies that kill cancer cells without affecting normal cells is the ultimate mode of treating cancers. The VP3, an avian virus-derived protein, can specifically initiate cell death through several signal transduction pathways leading to apoptosis. In cancer, chemoresistance and cell survivability implicate the cell surface protein, CD147.

**Methods:**

In this study, transfection of VP3 and silencing of CD147 genes was achieved through the treatment of tumors with pVIVO1-GFP/VP3 (VP3), psiRNA-CD147/2 (shCD147/2), and their combination of CT26 colon cancer cell-induced in mice. The effectiveness of tumor-treatment was ascertained by electrophoresis, TUNEL assay, and flow cytometry analysis. While histopathological and biochemical analysis were used as toxic side effect identification.

**Results:**

The tumor growth delay index (TGDI) after treatment with VP3, shCD147/2, and their combination treatments increased by 1.3-, 1.2-, 2.0- and 2.3-fold respectively, over untreated control. The VP3-shCD147/2 combination treatment was more efficacious then either VP3 or shCD147/2 alone in the retardation of mouse CT26 colorectal cell tumor allograft.

**Conclusion:**

The antitumor effect of the combination treatment is the result of synergistic effects of VP3 and shCD147/2 on the tumor cells resulting in apoptosis. Thus, the study shows that combination of VP3 and shCD147/2 treatment can be developed into a potential approach for anticolorectal cancer treatment regimen.

**Electronic supplementary material:**

The online version of this article (doi:10.1186/s12885-016-2530-8) contains supplementary material, which is available to authorized users.

## Background

Colorectal cancer is the third most common cancer cases after lung (1.82 million) and breast (1.67 million) cancers [1]. Within the next 15 years approximately 1.4 million new cases of colorectal cancer are expected to occur with an estimated death of 693 000 that would account for 8.5 % of all cancer deaths [2]. Poor survival rate of colon cancer patient is partly due to poor understanding of the disease and its progression, invasion, migration and metastasis [3].

Basigin/CD147, a transmembrane glycoprotein of the immunoglobulin superfamily, is expressed widely on many cell types and highly expressed in various tumor cells [4]. CD147 play an important role in proliferation, angiogenesis, invasiveness and metastatic activity of malignant melanoma [5]. Increased expression of CD147 was shown to correlate with enhanced tumor progression and poor prognosis in different cancers [6–8]. Thus, an attractive way to curb tumor progression is through suppression of the CD147-dependent cell proliferation, invasion and metastasis by RNAi-mediated silencing [5, 9] and eventually induce cell apoptosis due to detachment of anchorage-dependent cell from the surrounding extracellular matrix [10, 11].

In the development of anti-cancer compounds, apoptosis is the preferred mode of cancer cell death. Viral protein of the avian anemia VP3/Apoptin has a positively charged C-terminus that is reported to induce apoptosis selectively on transformed and tumor cells, leaving normal cells intact [12, 13]. VP3 in the murine tumor model was shown to be anti-tumorigenic, mostly through induction of apoptosis [14, 15]. The ability of VP3 in inducing p53-independent apoptosis has been demonstrated in more than 70 tumor cell lines [16]. Although simultaneous VP3, interleukin-24 [17], interleukin-18 [18], upregulations and survivin downregulation [19] seemed to show greater anti-tumor activity than VP3 alone, the combined effect of VP3 and shRNA on CD147 affecting tumor growth and progression is yet to be investigated. In this study the combined effect of pVIVO1-GFP/VP3 and psiRNA-CD147/2 was examined in the attempt to discover a new therapeutic approach for colorectal cancers.

## Methods

### Animals

Female, 5 to 6 week-old BALB/c mice were obtained from Institute Medical Research (IMR, Malaysia) and were acclimatized for a week prior to use. All mice were kept in individually ventilated cages (IVC) with constant rotation rate of 70 air-changes/h. Mice were fed on sterilized commercial diet, given water *ad libitum* and subjected to 12 h light and dark cycle. The study was performed with approval of the Institutional Animal Care and Use Committee, Universiti Putra Malaysia (UPM/FPV/PS/3.2.1551/AUP-R103).

### Tumor cells

Murine CT26 colon cancer cell lines (ATCC® CRL-2638™) was purchased from American Type Culture Collection (ATCC) and cultured in RPMI 1640 medium (Gibco, USA) supplemented with 10 % heat inactivated fetal bovine serum (FBS) (Gibco, USA) and 1 % Penicillin/Streptomycin antibiotic solution (Gibco, USA).

### Plasmid DNA

The psiRNA-CD147/2 was constructed by cloning short hairpin RNA (shRNA) specifically targeting mouse CD147 mRNA (GenBank: NM_001077184) into the eukaryotic expression vector, psiRNA-h7SKzeo (InvivoGen, USA) equipped with h7SK promoter region. shCD147/2 nucleotides were designed using siRNA Wizard software (http://www.invivogen.com/sirnawizard/): sense 5’-GTACCTCGGCAATCACCAATAGCACTGATCAAGAGTCAGTGCTATTGGTGATTGCCTTTTTGGAAA-3’ and antisense 5’-AGCTTTTCCAAAAAGGCAATCACCAA TAGCACTGACTCTTGATCAGTGCTATTGGTGATTGCCGAG-3’. The oligonucleotides were annealed and cloned into *Acc 65I* and *HindIII* sites of the vector according to manufacturer’s instruction. The construction of plasmid pVIVO1-GFP/VP3 was described previously [20]. Briefly, pVIVO1-GFP/VP3 contains VP3 gene under the control of CMV enhancer and GRP78 promoter region. VP3 gene was synthesized from a local Chicken Anaemia Virus isolate (GenBank: AF_030518). The unmodified psiRNA-h7SKzeo and pVIVO1-GFP/LacZ were used as controls treatment. All plasmids DNA used were purified with Qiagen columns (Qiagen, Germany) using endotoxin-free reagents according to the manufacturer’s protocol. Plasmid DNA was diluted in sterile PBS, left at room temperature for 10 min prior to intratumoral injection.

### Animal colon cancer model

The mice were anesthetized with 40 mg ketamin plus 8 mg xylazine/kg bwt intraperitoneally and placed on 37 °C warming pad. Cell suspension containing 1 × 10^6^ CT26 cells in 0.2 mL sterile PBS were subcutaneously injected on the right flank of the mice with minimal trauma. The mice were observed on alternate days for tumor development and palpable tumors were measured. Treatments of the mice were instituted when the tumors reached sizes of approximately 50 mm^3^ or ≥200 mm^3^. Each control and treatment group comprise of 3 mice.

### Measurement of tumor growth and evaluation of antitumoral effect

Tumor volume was determined by measuring the greatest length and width using calipers, and calculated by using the following formula [21]:

$$ \mathrm{Tumor}\ \mathrm{volume}\ \left(\mathrm{V}\right) = \left(\mathrm{length} \times \mathrm{widt}{\mathrm{h}}^2\right)/2 $$

Evaluation of antitumoral effect was determined according to Sanceau J. *et al.* [22]. Individually relative tumor volume (RTV) was defined as follows:

$$ \mathrm{Relative}\ \mathrm{tumor}\ \mathrm{volume}\ \left(\mathrm{R}\mathrm{T}\mathrm{V}\right) = {\mathrm{V}}_{\mathrm{x}}/{\mathrm{V}}_1 $$

where V_x_ is the volume (mm^3^) at a specific time and V_1_ is the volume at the beginning of treatment. Treatment efficacy was expressed as the percentage of tumor growth inhibition (TGI) as follows:

$$ \mathrm{T}\mathrm{G}\mathrm{I}\ \left(\%\right)=100\hbox{-} \left(\mathrm{T}/\mathrm{C} \times 100\right) $$

where T and C is the mean RTV of treated and control group at the time of sacrifice, respectively. Tumor growth delay (TGD) was determined as the time required for the tumor volume to reach 10-fold over the initial volume. Tumor growth delay index (TGDI) was calculated as follows:

$$ \mathrm{TGDI}=\mathrm{T}\mathrm{G}{\mathrm{D}}_{\mathrm{T}}/\mathrm{T}\mathrm{G}{\mathrm{D}}_{\mathrm{C}} $$

where TGD_T_ and TGD_C_ is the mean TGD of the treated and control group, respectively.

### Experimental design

#### Protocol I: Individual treatment

When the tumors reached volumes of 45 to 50 mm^3^, each mouse was treated intratumorally with 100 μg of treatment in 70 μl of sterile PBS. Three doses of treatment were injected at alternate days into established CT26-tumors according to the following regimens: (a) Control group (mock treatment) - 100 μg of either pVIVO1-GFP/LacZ (LacZ) or psiRNA-h7SKzeo (zeo), (b) Treatment 1: 100 μg pVIVO1-GFP/VP3 (VP3), (c) Treatment 2: 100 μg psiRNA-CD147/2 (shCD147/2). Tumors growth were measured on alternate days for 20 days post-treatment.

#### Protocol II: Combination treatment

Mice with tumor size of ≥200 mm^3^ were treated intratumorally with 100 μg of treatment in 70 μl of sterile PBS according to the following regimens: Control groups were either a) non-treated, b) received 3 doses of 100 μg pVIVO1-GFP/LacZ or c) 3 doses of 100 μg psiRNA-h7SKzeo. Treatment mice received either a) 3 doses of 100 μg of pVIVO1-GFP/VP3 or b) 3 doses of 100 μg of psiRNA-CD147/2. In combination therapy, mice received either a) 3 doses of 50 μg of pVIVO1-GFP/VP3 in combination with 3 doses of 50 μg of psiRNA-CD147/2 or b) 3 doses of 100 μg of pVIVO1-GFP/VP3 in combination with 3 doses of 100 μg of psiRNA-CD147/2, representing low and high dose treatments, respectively. In combination study, mice received pVIVO1-GFP/VP3 and psiRNA-CD147/2 treatments alternately, while in control and single treatment, mice received doses at alternate days. Tumor growth was examined on alternate days for 25 days post-treatment.

Blood was collected from all mice prior to sacrifice and tumor tissues were fixed either in 10 % neutral buffered formalin for hematoxylin and eosin staining and immunohistochemical analysis or flash-frozen in liquid nitrogen and stored at -80 °C for molecular analysis. The serum creatinine, blood urea nitrogen (BUN), alkaline phosphatase (ALP), alanine transaminase (ALT) and aspartate transaminase (AST) were determined spectrophometrically using standard commercial kits (Roche, Swizerland).

### DNA fragmentation analysis

In this analysis the genomic DNA (gDNA) from frozen tumor tissues were isolated using DNAzol (Molecular Research Centre, Inc, USA) in accordance with manufacturer’s protocol. Briefly, 50 mg of tumor tissue was rinsed with PBS. DNAzol-tumor tissue homogenization was performed using pre-cleaned pestle and mortar. The homogenate was then centrifuged for 10 min at 10 000 × g to sediment the remaining insoluble tissues. Thereafter, the viscous supernatant was transferred to new microcentrifuge tube and apoptotic DNA fragments were precipitated using 100 % absolute ethanol at 6 000 × g for 6 min. After centrifugation, the DNA pellet was rinsed 2 times with 70 % ethanol by inverting a few times and sediment at 1000 × g for 1 min. Finally, DNA pellet was air dried and resuspended in sterile dH_2_O. DNA fragmentation was determined by 2 % agarose gel electroporation in 1 × TBE buffer and run at 80 V for 45 min. The DNA was stained with ethidium bromide and visualized under UV transilluminator. Apoptotic cells were appeared as a ladder pattern while necrotic as a smear pattern on the gel. Intact genomic DNA appeared as a band at the top of the lane.

### Terminal deoxynucleotidyl transferase-mediated nick end-labeling assay

Apoptotic endonucleases cleave DNA to produce fragments with 3’-OH groups that can be detected on tumor sections stained with FragEL DNA Fragmentation Detection Kit-Klenow Enzyme (Calbiochem, USA) and recorded digitally using light microscopy (Nikon Elipse TE2000-S, Nikon, Japan) at × 200 magnification. Approximately, 5–10 random images were taken for each group (n = 3). Briefly, fixed tumor tissues were dehydrated, cleared, infiltrated and paraffin embedded. Tissue sections of 4 μm were prepared using rotary microtome and mounted onto glass slides, deparaffinized, rehydrated and treated according to manufacturer’s procedure. Apoptosis was determined by stained nuclei with brown color after labelled with DAB. Tumor sections were counterstained with methyl green. TUNEL-positive cells were counted and analyzed using Image J software (ImageJ 1.43u, USA), and the apoptotic index (AI) was calculated as percentage of TUNEL-positive cells per total number of cells.

### Flow cytometry

To further ascertain that the treatment caused tumor cell death through apoptosis rather than necrosis, the tumor cells were subjected to flow cytometry after staining with annexin V-FITC and propidium iodide. The technique allows for differentiation between living, apoptotic, and necrotic cells. Apoptotic cells were further differentiated into those in early and late apoptosis. This method detects the translocation of the negatively charged phospholipid phosphatidylserine (PS) on cell membrane surface during the early stages of apoptosis. Single cell suspensions were subjected to flow cytometry following Annexin V-FITC and propidium iodide (PI) staining using the ApopNexin™ FITC Apoptosis Detection Kit (Chemicon, USA). For single cell preparation, tumor tissues were placed on sterile petri dish and washed 3 times with PBS. Tumor tissues were cut into small pieces (1–2 mm^3^ in size) and then carefully disintegrated with fine forceps in 2 ml of PBS. Cells were then transferred into a 15 ml conical centrifuge tube and resuspended gently and rapidly in 10 ml of ice-cold PBS. The cells suspension was then centrifuged at 170 × g for 1 min (4 °C) to sediment the remaining tissue fragments. The supernatant containing single cells was transferred into a new 15 ml conical centrifuge tube and centrifuged at 170 × g for 10 min. The supernatant was discarded and pellet was resuspended in ice-cold PBS at a concentration of 1 × 10^6^ cells/mL and kept on ice. Then, tumor cells suspensions were centrifuged to remove PBS and resuspended in ice-cold 1× Binding Buffer (10 mM Hepes/NaOH; pH 7.4, 140 mM NaCl and 2.5 mM CaCl_2_) at 1 × 10^6^ cells/mL. 200 μL of cells suspension were aliquoted in polystyrene round-bottom tube and stained with 3 μL of Annexin V-FITC. 2 μL of 100× PI solutions were added to the Annexin V-FITC-labelled cells and the suspension incubated at room temperature in the dark for 15 min and analyzed immediately using the Becton Dickinson FACS Calibur equipped with CellQuest Pro software. Cells labelled as FITC^+^/PI^−^ are in early apoptosis; cells labelled as FITC^−^/PI^+^ are necrotic or broken; cells labelled as FITC^+^/PI^+^ are either in late apoptosis or secondary necrosis; and cells negatively labelled as FITC^−^/PI^−^ are viable.

### Statistical analysis

The results are expressed as mean ± standard error of the mean. The data were analyzed by either Student’s paired *t*-test or ANOVA followed by Tukey multiple comparison *post hoc* test. The *P* value of <0.05 was considered significant.

## Results

### Colon cancer mice model

A small tumor mass of 48.41 ± 1.28 mm^3^ was palpable at day 10 day post-implantation. The tumor was allowed to grow achieving a size of 210.9 ± 7.26 mm^3^ by 15 days post-implantation.

### Complete regression by VP3 or shCD147/2 treatment in small size tumor

**Fig. 1:**
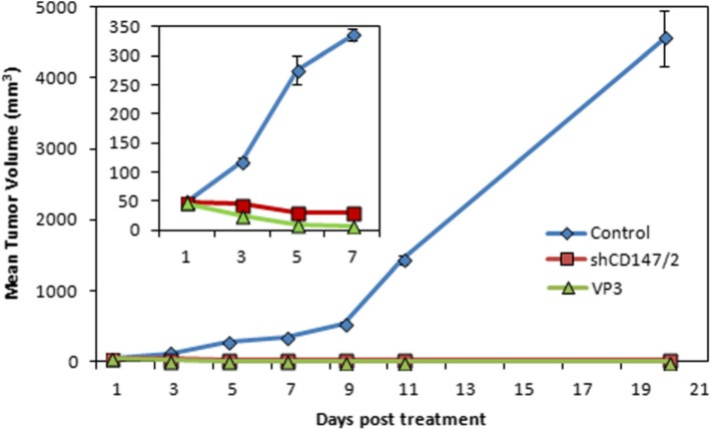
Development and treatment of CT26 colon cancer in mice. The tumors were treated with VP3 or shCD147/2 and tumor volume was determined on alternate day for 20 days. The treatments effectively suppressed tumor growth. Inset depicting zoom-in of days 1-7

In mice with tumor size approximately 50 mm^3^, VP3 as well as shCD147/2 treatments showed regression in tumor volume from 47.0 ± 3.5 and 48.4 ± 2.2 mm^3^ before treatment to 7.1 ± 1.1 and 31.1 ± 7.5 mm^3^ on the day 7 post-treatment, respectively (Fig. 1). By that time the tumor volume in untreated control mice increased to 336.5 ± 9.2 mm^3^ and continued to increase rapidly reaching a volume of 4 000 mm^3^ by day 20. In mice treated with either VP3 or shCD147/2, the tumor remained smaller than 50 mm^3^ and finally regressed completely by day 30 post-treatment.

### VP3-shCD147/2 combination treatment increased tumor growth delay index (TGDI)

**Fig. 2:**
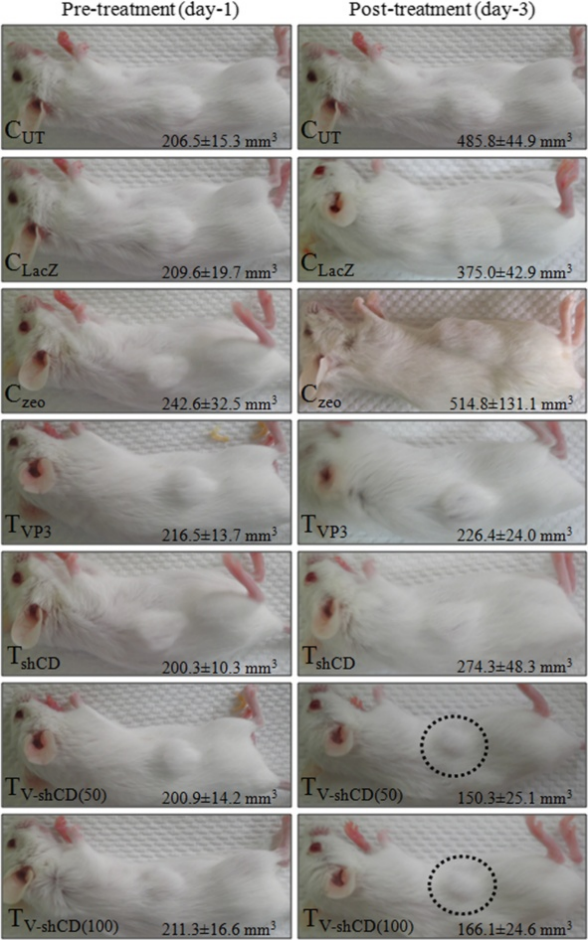
Representative photographs of tumors taken at day-1 before treatment and day-3 after treatment. C_UT_ = Untreated control, C_LacZ_ = pVIVO1-GFP/LacZ control, C_zeo_ = psiRNA-h7SKzeo control, T_VP3_ = Treated with pVIVO1-GFP/VP3 (VP3), T_shCD_ = Treated with psiRNA-CD147/2 (shCD147/2), T_V-shCD(50)_ and T_V-shCD(100)_ = Treated with low and high dose of VP3-shCD147/2 combination respectively. Circles show significantly size reduction in combinative treatment

In mice with tumor size of ≥200 mm^3^, both low and high dose VP3 plus shCD147/2 combination treatments caused more significant (p < 0.05) antitumor effects than either VP3 or shCD147/2 alone (Fig. 2). In combination treatments, the tumor size decreased from the initial size of 200.9 ± 14.2 and 211.3 ± 16.6 mm^3^ to 150.3 ± 25.1 and 166.1 ± 24.6 mm^3^ on day 3 post-treatment for low and high dose, respectively. Treatments with either VP3 or shCD147/2 did not reduce tumor size, on day 3. In fact the tumor increased slightly in size from 216.5 ± 13.8 to 226.4 ± 24.0 mm^3^ and 200.3 ± 10.4 to 274.3 ± 48.3 mm^3^ for VP3 and shCD147/2 treatment, respectively.

**Fig. 3:**
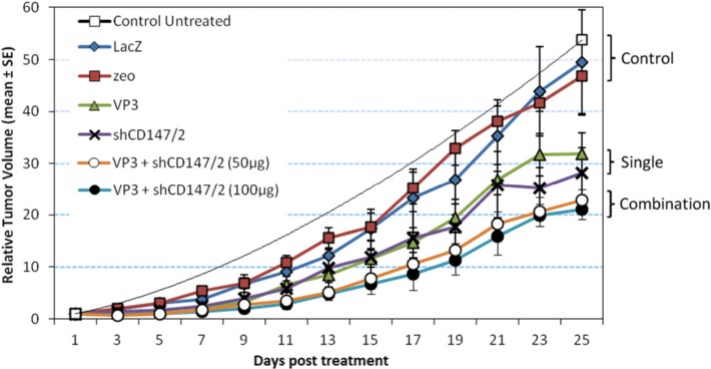
Relative tumor volumes in mice CT26 colon cancer cell line-induced tumor treated with LacZ, zeo, VP3, shCD147/2 and VP3-shCD147/2 combination. On long-term, combination VP3-shCD147/2 treatments were effective in inhibiting tumor growth than either VP3 or shCD147/2 alone. The plasmids used in the treatments were pVIVO1-GFP/LacZ (LacZ), psiRNA-h7SKzeo (zeo), pVIVO1-GFP/VP3 (VP3) and psiRNA-CD147/2 (shCD147/2)

Relative tumor volume, which is the size of tumor at a given time compared with the initial size, is shown in Fig. 3. The growth began slowly and began to accelerate from approximately 12 days post-treatment. However, in untreated mice the tumor grew rapidly reaching much higher relative volume than in treated mice at day 25 post-treatment. Tumors treated with VP3, shCD147/2, low and high dose combination undergo 40.0, 45.2, 51.1 and 60.3 % of growth inhibition, respectively. TGDI, indicating treatment efficiency is calculated as the delay in days taken by the treated tumors to reach a 10-fold RTV divided by the delay in the control group. The TGDI of tumors treated with VP3, shCD147/2, low and high dose combination treatments increased by 1.3-, 1.2-, 2.0- and 2.3-fold respectively, from the initial volume. Thus, the results showed that combination VP3 and shCD147/2 treatments were more effective than VP3 or shCD147/2 alone.

### Biochemical analysis of colon cancer mice model after treatment

**Fig. 4:**
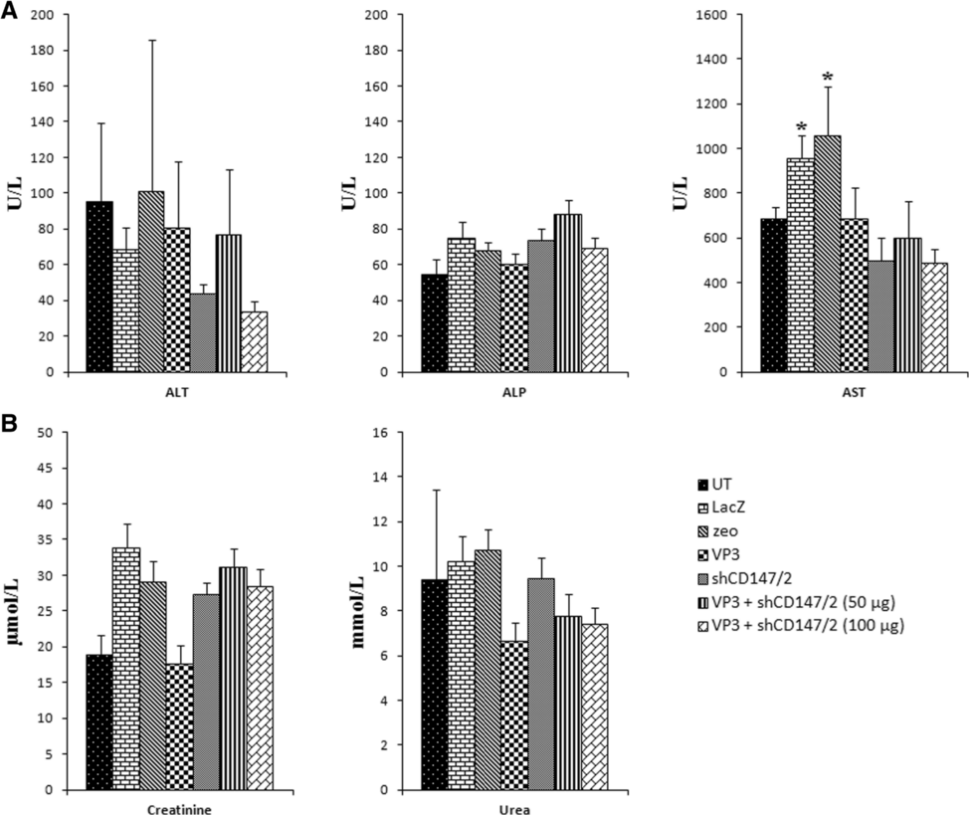
Serum liver enzymes and kidney function parameters in treated mice with CT26 colon cancer cell-induced tumor. **a** Liver enzymes; ALT = alanine transaminase, ALP = alkaline phosphatase, AST = aspartate transaminase and **b** kidney function parameters; creatinine and urea. Data are mean ± SEM. *P < 0.05 compared to the untreated group. UT = Untreated control, LacZ = pVIVO1-GFP/LacZ, zeo = psiRNA-h7SKzeo, VP3 = pVIVO1-GFP/VP3 and shCD147/2 = psiRNA-CD147/2

Serum liver enzymes and kidney function parameter concentrations were estimated to determine safety of plasmids pVIVO1-GFP/VP3 (VP3) and psiRNA-CD147/2 (shCD147/2) as therapeutic compounds. Except for AST, neither liver enzymes nor kidney function parameters showed significant (p > 0.05) difference between treatments and untreated control (Fig. 4). However, AST, which in this case reflects muscle integrity were significantly (p < 0.05) higher in mice tumor treated with LacZ and zeo.

### VP3 overexpression and knockdown of CD147 induced a cellular morphologic change in CT26 tumor cells

**Fig. 5:**
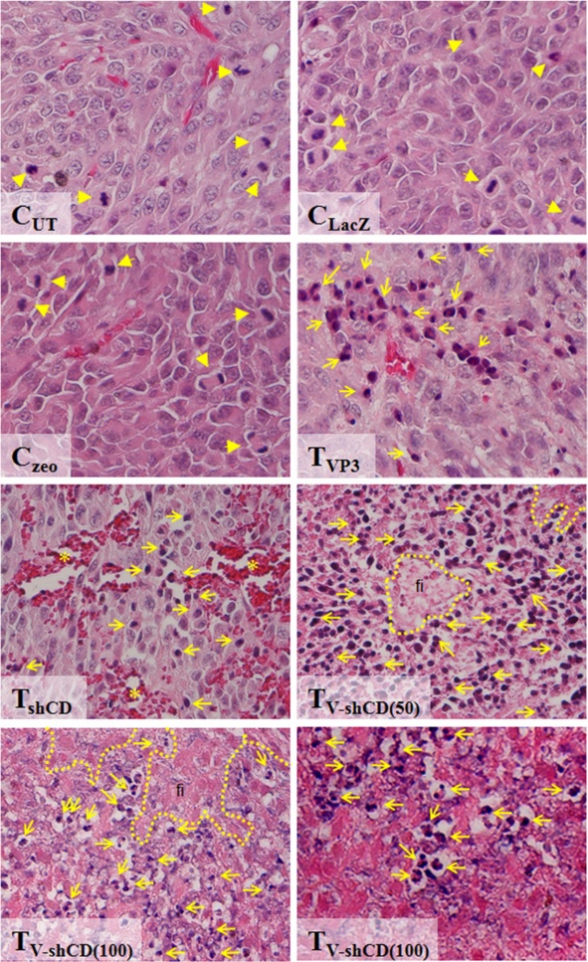
Histopathology of CT26 colon cancer cell line-induced tumor in mice. C_UT_ = Untreated control, C_LacZ_ = pVIVO1-GFP/LacZ control, C_zeo_ = psiRNA-h7SKzeo control, T_VP3_ = Treated with pVIVO1-GFP/VP3 (VP3), T_shCD_ = Treated with psiRNA-CD147/2 (shCD147/2), T_V-shCD(50)_ and T_V-shCD(100)_ = Treated with low and high dose of VP3-shCD147/2 combination respectively. Tumors were resected at day 25 post-treatment. Sections showing numerous mitotic features (arrow heads), extensive fibrosis (fi border by a dotted line), blood vessel ruptures (asterisk) and numerous apoptotic bodies (full arrows). H&E (×200). Right panel of T_V-shCD(100)_ at × 400 magnification

Under H&E staining, tumor tissues in all mice treated with VP3, shCD147/2 or their combination showed typical features of apoptosis to include interstitial spaces, apoptotic bodies, and dark nuclei. In contrast, control, LacZ- and zeo-treated mice did not show similar cellular morphology. Tumor of treated mice also showed fewer mitotic events than those of the controls. Tissue section from tumors treated with shCD147/2 also showed numerous blood vessel ruptures (Fig. 5).

### VP3, shCD147/2 and combinations treated tumors characterized by DNA laddering

**Fig. 6:**
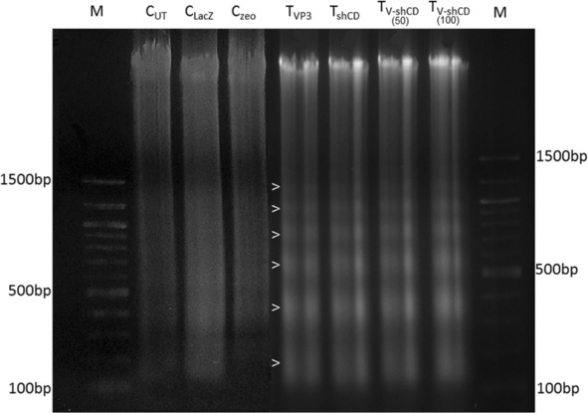
DNA fragmentation on 2.0 % agarose gel. Lanes M: DNA marker (NEB, USA); C_UT_ = Untreated control, C_LacZ_ = pVIVO1-GFP/LacZ control, C_zeo_ = psiRNA-h7SKzeo control, T_VP3_ = Treated with pVIVO1-GFP/VP3 (VP3), T_shCD_ = Treated with psiRNA-CD147/2 (shCD147/2), T_V-shCD(50)_ and T_V-shCD(100)_ = Treated with low and high dose of VP3-shCD147/2 combination respectively. Open arrow heads showing the laddering pattern

Treatment with VP3, shCD147/2 or their combination at 72 h post-treatment lead to high intensity laddering indicating apoptotic activity (Fig. 6). This observation was most obvious in tumors treated with 100 μg VP3-shCD147/2 combination. The gels from untreated control, LacZ- and zeo-treated tumors showed smeared bands suggesting complete DNA lysis indicating necrosis.

### Enhanced apoptotic events in VP3-shCD147/2 combination treated tumors

**Fig. 7:**
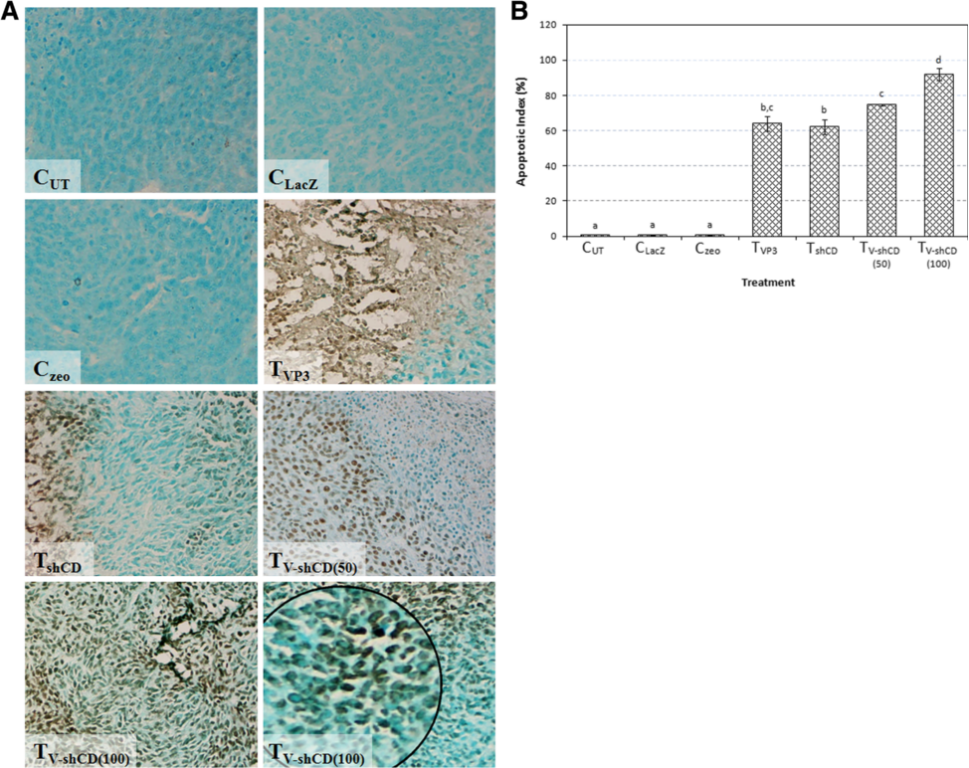
Apoptosis in treated mice CT26 colon cancer cell-induced tumor determined by TUNEL assay at day 25 post-treatment. **a** Tumor tissue with nick-labeling fragmented DNA. Apoptotic cells are stained dark brown while viable cells stained green color, 200×. Magnifying: shows brownish to darkish labeled nuclear granules. **b** Apoptotic index of tumor tissue. Error bars represent the standard error of the mean. Means with different letters are significantly (p < 0.05) different. C_UT_ = Untreated control, C_LacZ_ = pVIVO1-GFP/LacZ control, C_zeo_ = psiRNA-h7SKzeo control, T_VP3_ = Treated with pVIVO1-GFP/VP3 (VP3), T_shCD_ = Treated with psiRNA-CD147/2 (shCD147/2), T_V-shCD(50)_ and T_V-shCD(100)_ = Treated with 50 and 100 μg dose of VP3-shCD147/2 combination respectively

Apoptotic endonucleases cleave nuclear DNA to produce fragments with 3’-OH groups that can be detected on tumor sections. Apoptotic cells were observed as dark brown nuclear staining while viable cells stained green color (Additional file 1: Figure S1). Tumors treated with VP3, shCD147/2 and their combinations showed numerous TUNEL-positive cells indicating apoptosis (Fig. 7a). There were more than 60 % apoptosis in the treated tumors compared to <1 % in the untreated and control tumors. The apoptotic index (AI) of tumors treated with VP3 and shCD147/2 were 63.9 ± 4.0 and 62.1 ± 4.2 % respectively, while in the 50 and 100 μg VP3-shCD147/2 combination treatments, the AI was 74.7 ± 0.4 and 92.1 ± 3.5 % respectively (Fig. 7b).

### VP3-shCD147/2 combination treatment increased the rate of apoptosis in CT26 tumor cells

**Fig. 8:**
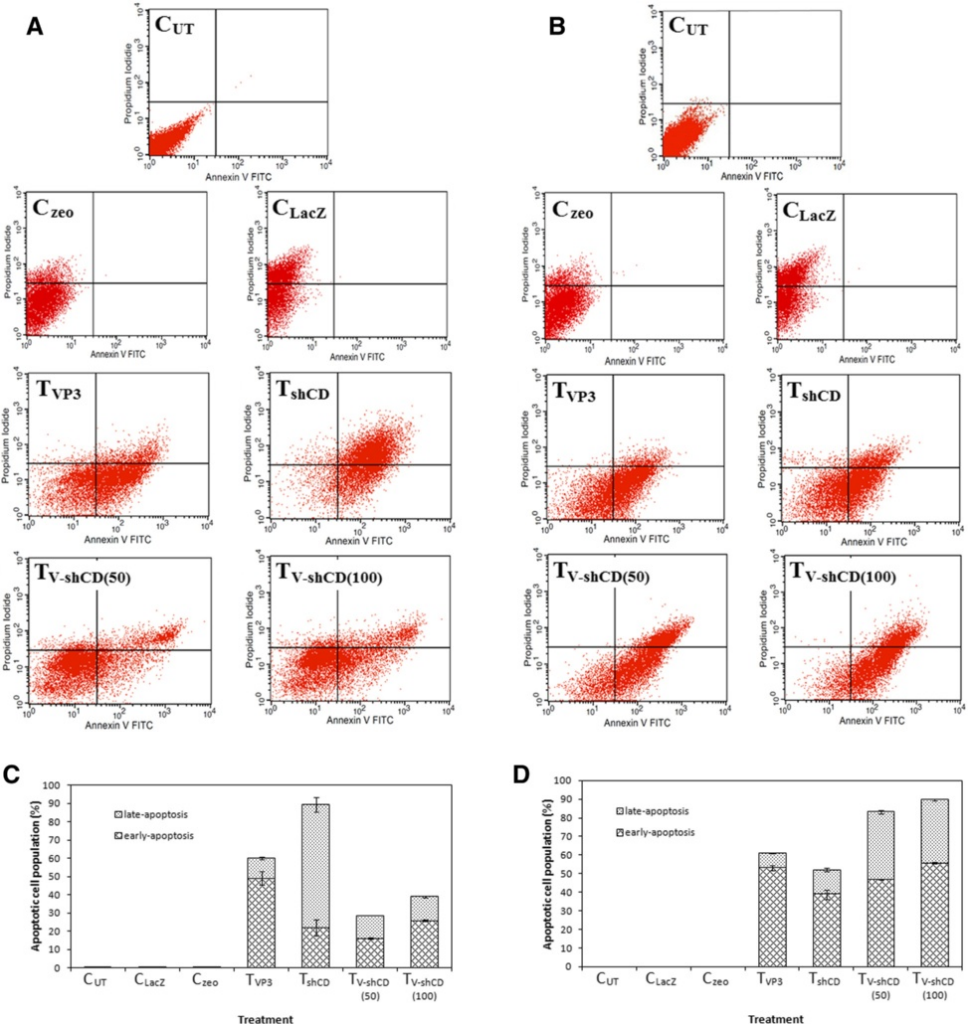
Apoptosis in treated mice CT26 colon cancer cell-induced tumor determined by fluorescence-activated cell sorting. Tumor cells were labeled with FITC-annexin V and propidium iodide at **a** day 3 post-treatment and **b** day 25 post-treatment. Histogram showed quantification of apoptosis in treated tumors at **c** day 3 post-treatment and **d** day 25 post-treatment. C_UT_ = Untreated control, C_LacZ_ = pVIVO1-GFP/LacZ control, C_zeo_ = psiRNA-h7SKzeo control, T_VP3_ = Treated with pVIVO1-GFP/VP3 (VP3), T_shCD_ = Treated with psiRNA-CD147/2 (shCD147/2), T_V-shCD(50)_ and T_V-shCD(100)_ = Treated with 50 and 100 μg dose of VP3-shCD147/2 combination respectively

Figure 8a, b shows proportion in percentage of live viable, necrotic, early apoptotic and late apoptotic cells in untreated, control LacZ, control zeo, VP3, shCD147/2, 50 and 100 μg combination treated tumor at day-3 and day-25 respectively. The rate of apoptosis in mice tumor treated with shCD147/2 on day 3 post-treatment was 89.59 ± 5.85 %, while in tumors treated with VP3, 50 and 100 μg combination treatments were 60.10 ± 3.98, 28.62 ± 0.47 and 39.05 ± 0.56 %, respectively (Fig. 8c). The rate of apoptosis in VP3-treated tumors remained constant till day 25 post-treatment. In shCD147/2-treated tumors the rate of apoptosis decreased to 52.07 ± 2.65 % and in 50 and 100 μg combination treatments the apoptosis rate increased to 83.14 ± 0.99 and 89.51 ± 0.56 %, respectively (Fig. 8d).

## Discussion

In this study, transfection of colorectal tumors with VP3 gene in combination with psiRNA-CD147/2-induced CD147 silence as cancer gene therapy for colorectal cancers in the CT26 colorectal cancer cell-induced mouse model was investigated. The CT26 cell line is a rapid-growing grade IV carcinoma that can readily undergo metastasis [23]. For that reason, the CT26 mouse tumor is one of the most extensively used model in the investigation of colorectal carcinomas [24]. Intra-tumoral administration of pVIVO1-GFP/VP3 was shown to cause significant reductions in tumor size in these mice [25, 26]. The viral-vectored VP3 has also been shown to cause regression and complete remission of the xenograft of human hepatomas grown in mice [27].

To determine the therapeutic effect of VP3 protein on CT26 tumors, a sustainable and tumor-inducible GRP-promoter was used to enhance the VP3 expression in a targeted cell population [28–30]. When the tumor was treated with recombinant pVIVO1-GFP/VP3 there was complete regression of tumor showing that recombinant plasmids harboring VP3 can be anti-tumorigenic. Partial regression of CT26 tumors can be induced by CD147 silencing as shown in mice with human colon cancer xenograft [31] and can be achieved with psiRNA-CD147/2. Although pVIVO1-GFP/VP3 and psiRNA-CD147/2 are both effective antitumor agents, our study showed that pVIVO1-GFP/VP3 is superior to psiRNA-CD147/2. Further, when pVIVO1-GFP/VP3 was used in combination with psiRNA-CD147/2, the antitumor effect was enhanced. This observation suggests that pVIVO1-GFP/VP3 and psiRNA-CD147/2 act synergistically in causing tumor regression. It is proposed that the synergistic effect is attributed to the tumor CD147 silencing causing inhibition of tumor cell proliferation and invasion, and proapoptotic VP3 gene transfected into the tumor cells through the use of pVIVO1-GFP/VP3 [32].

Induction of apoptosis is the mode of cell death targeted by most antitumor agents. Treatments with pVIVO1-GFP/VP3, psiRNA-CD147/2 and their combination were shown to cause apoptosis of CT26 mouse tumor cells. The antitumor effect of pVIVO1-GFP/VP3 and psiRNA-CD147/2 was rapid and remained constant for the period of the study in the case of pVIVO1-GFP/VP3 treatment or eventually waned when psiRNA-CD147/2 was used. When pVIVO1-GFP/VP3 and psiRNA-CD147/2 were administered as combination treatment, apoptosis of tumor cells was slow to occur; however, after 25 days the combination in fact killed the majority of tumor cells. The mode of tumor cell death was apoptosis and this was supported by histopathology, where tumor tissues showed abundance of apoptotic features. On the contrary, there was an abundance of mitotic features in the untreated and control tumor tissue indicating rampant tumor growth. One of the effects of psiRNA-CD147/2 is the triggering of indirect endothelial damage in the tumor tissues causing collapse of tumor vasculature. The net effect is poor blood flow and deprivation of oxygen supply to the tumors tissue culminating in tumor cell death.

The effectiveness of tumor-treatment was also ascertained by electrophoresis, TUNEL assay, and flow cytometry analysis. Normally, apoptotic DNA cleavage produced a signature pattern with high and low molecular weight fragments [33–35]. The presence of multiples of 180 to 200 bp DNA fragments indicated that treatment with pVIVO1-GFP/VP3, psiRNA-CD147/2 and their combination had caused apoptosis of tumor cells. CD147 knockdown eventually sensitized tumor cells to anoikis, which is a form of apoptosis induced by the detachment of anchorage-dependent cells from the surrounding extracellular matrix [10, 36]. In this study, the intensity of DNA ladder of the tumor cells treated with pVIVO1-GFP/VP3 was equivalent to that produced by those treated with psiRNA-CD147/2. However, the DNA ladder intensity was higher in tumors treated with pVIVO1-GFP/VP3-psiRNA-CD147/2 combination.

Another method used to assess for apoptosis is the *in situ* terminal deoxynucleotidyl transferase biotin-dUTP nick end labeling assays (TUNEL). The TUNEL assay was purposely used to detect nuclear DNA fragmentation by identifying generation of nicks and breaks in the DNA strands [37]. On the other hand, this assay was used in quantifying and comparing the number of TUNEL-positive cells between tumor samples; VP3-, shCD147/2- and combination-treated. The highest extent of apoptosis indicate by AI was in tumors treated with pVIVO1-GFP/VP3-psiRNA-CD147/2 combination compared to tumors from VP3 or shCD147/2 treated alone.

Flow cytometry analysis allowed the sensitive detection of apoptotising cells. The apoptosis percentage in the pVIVO1-GFP/VP3 treated tumor was sustained at day-3 and day-25 due to the GRP promoter sustainable effect. Meanwhile, apoptosis percentage in the psiRNA-CD147/2 treated tumor was high at day-3 and then declining at day-25 because vasculature rupture at the first few days causes anoikis which is interpreted as late-apoptosis; however the treatment becomes less effective with time. The apoptosis percentage in the combinatively pVIVO1-GFP/VP3-psiRNA-CD147/2 treated tumor was markedly increased compared to individually treated samples at day-25 post-treatment. The effects of shCD147/2 are triggering indirect damage to the pre-existing tumoral endothelium, results in collapse of the vasculature inside solid tumors. Thus, the tumor cells is deprived of oxygen supply or blocked from blood flow, which consequently leads to enhancement of pro-apoptosis induction by pVIVO1-GFP/VP3. The pVIVO1-GFP/VP3-psiRNA-CD147/2 combination treatment seemed to synergise the effects of pVIVO1-GFP/VP3 and psiRNA-CD147/2 by intensifying antitumor effect in prolonged treatment. Tumor growth is exponential in the early stages, then becomes less aggressive, and plateaus at the late stages of the disease [38]. Since psiRNA-CD147/2 is very effective early and pVIVO1-GFP/VP3 has a consistent effect throughout tumor development, the pVIVO1-GFP/VP3-psiRNA-CD147/2 combination treatment would be the more efficacious antitumor regimen than either pVIVO1-GFP/VP3 or psiRNA-CD147/2 alone.

Chemotherapy is plagued with side-effects, thus new drugs or therapeutic regimens require toxicity testing. In this study, the effect of pVIVO1-GFP/VP3, psiRNA-CD147/2 and their combination on the liver and kidneys were ascertained by determining serum ALT, ALP, AST, urea and creatinine concentrations. With the exception of slightly elevated AST concentration, all blood biochemical parameters were normal indicating that the liver and kidneys were not affected by the treatments. Increase in AST may be associated with some muscle damage or increased muscular activities that are not associated with the toxic effect of the treatments. Thus, pVIVO1-GFP/VP3, psiRNA-CD147/2 or their combination is generally nontoxic and safe to be in mice, but proper Pharmacokinetics (PK) analyses are required to confirm prior to clinical work in humans.

## Conclusions

The antitumor effect of the combination treatment is the result of synergistic effects of VP3 and shCD147/2 on the tumor cells resulting in apoptosis. Based on these studies, we conclude that the pVIVO1-GFP/VP3-psiRNA-CD147/2 combination therapy is potentially effective and safe regimen for treating colorectal cancers.

## Abbreviations

AI, apoptotic index; ALP, alkaline phosphatase; ALT, alanine transaminase; AST, aspartate transaminase; BUN, blood urea nitrogen; CD147, cluster of differentiation 147; gDNA, genomic deoxyribonucleic acid; GRP, glucose regulate protein; PI, propidium iodide; PS, phospholipid phosphatidylserine; RTV, relative tumor volume; shCD147/2, short hairpin CD147/2; shRNA, short hairpin RNA; TGD, tumor growth delay; TGDI, tumor growth delay index; TGI, tumor growth inhibition; TUNEL, terminal deoxynucleotidyl transferase-mediated nick end-labeling assay; VP3, virus protein 3

## Additional file

Additional file 1: Figure S1. Morphological and histological features of CT26 tumor. A) Exposed CT26-induced tumor becoming a spheroid measuring 2–2.5 cm in diameter at day 25 post-treatment. The tumor mass is well-defined and vascularized. *Right panel*, cross section of the tumor. B) Photomicrographs of H&E-stained tumor sections. The tumor was dissected at day 25 post-treatment for histological analysis. *Top*, peripheral region showing intact tumor cells, and, *bottom*, inner region showing tumor cells and a necrotic core. 100× magnification. C) TUNEL assay were evaluated on CT26 tumor sections. Apoptotic cells indicate by TUNEL-positive are stained dark brown while viable cells stained green color. *Top*, peripheral region showing intact tumor cells and apoptotic cells, and, *bottom*, inner region showing apoptotic cells, viable tumor cells and a necrotic core. 100× magnification. (TIF 636 kb) (TIF 635 kb)
