# Supplementary figures and images for: Combination of VP3 and CD147-knockdown enhance apoptosis and tumor growth delay index in colorectal tumor allograft
Source: BMC Cancer. 2016 Jul 13;16:461. doi: 10.1186/s12885-016-2530-8 (PMC4944445; doi:10.1186/s12885-016-2530-8)

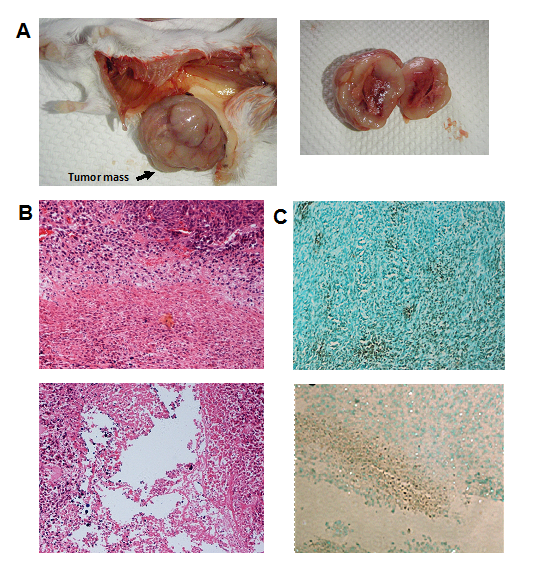

Supplement: Additional file 1: Figure S1. — Morphological and histological features of CT26 tumor. A) Exposed CT26-induced tumor becoming a spheroid measuring 2–2.5 cm in diameter at day 25 post-treatment. The tumor mass is well-defined and vascularized. Right panel, cross section of the tumor. B) Photomicrographs of H&E-stained tumor sections. The tumor was dissected at day 25 post-treatment for histological analysis. Top, peripheral region showing intact tumor cells, and, bottom, inner region showing tumor cells and a necrotic core. 100× magnification. C) TUNEL assay were evaluated on CT26 tumor sections. Apoptotic cells indicate by TUNEL-positive are stained dark brown while viable cells stained green color. Top, peripheral region showing intact tumor cells and apoptotic cells, and, bottom, inner region showing apoptotic cells, viable tumor cells and a necrotic core. 100× magnification. (TIF 636 kb) (TIF 635 kb) [file 12885_2016_2530_MOESM1_ESM.tif]
